# From hatching to juvenile: Larval development of *Vieja fenestrata* (Teleostei: Cichlidae)

**DOI:** 10.1111/jfb.15898

**Published:** 2024-08-10

**Authors:** Rubén Alonso Contreras‐Tapia, Marcela Ivonne Benítez‐Díaz Mirón, Gabriela Garza Mouriño, María Elena Castellanos‐Páez

**Affiliations:** ^1^ Laboratorio de Rotiferología y Biología Molecular de Plancton, Departamento El Hombre y su Ambiente Universidad Autónoma Metropolitana, Unidad Xochimilco Mexico City Mexico

**Keywords:** blackstripe cichlid, development, Herichthyini, Neotropical, ontogenetic

## Abstract

This study delves into the early development of *Vieja fenestrata* (Cichlidae), with a specific focus on the description of external morphological and morphometric changes, and growth patterns from hatching to the loss of larval characters under controlled laboratory conditions at a temperature of 28°C. Asynchronous hatching was observed between 58 and 60 h postfertilization, with the posterior body emerging first. Over 14 days, significant morphological, physiological, and behavioral changes were observed, revealing a complex developmental trajectory. The initial developmental phases were characterized by rapid vascularization, fin differentiation, and heightened activity, and the subsequent days witnessed the flexion of the notochord, emergence of swim bladder functionality, and transition to exogenous feeding. Maturation progressed with the absorption of the yolk sac, regression of cement glands, and fin ray development, culminating in metamorphosis by 14 days post‐hatching. Throughout this period, evolving pigmentation patterns and structural adaptations highlight the species' adaptive strategies. During the larval period of *V. fenestrata*, substantial changes in morphological proportions were observed. Before the inflection, tail length, trunk length, and body depth had negative allometric growth, and head length, eye diameter, and snout length had positive allometric growth. After the inflection, body depth and snout length showed positive allometric growth; head length and trunk length exhibited isometric growth, whereas tail length and eye diameter demonstrated negative allometric growth. These findings contribute insights into the intricate developmental dynamics of *V. fenestrata.* Moreover, further research may explore these developmental dynamics' ecological and evolutionary implications.

## 1 INTRODUCTION

The family Cichlidae comprises a highly diversified vertebrate group, totaling 1756 valid species (Fricke et al., 2024), and is of considerable ecological and socioeconomic importance. Cichlids have been the focus of attention in various research areas, including species delimitation, phylogenetic reconstruction, ecology, and ethology, and have been proposed as ideal model organisms for studying evolution, ecology, development, and behavior (Burress, 2015; Kocher, 2004; McMahan et al., 2015; Powder & Albertson, 2016; Streelman et al., 2003; Taborsky, 2016; Woltering et al., 2018). The detailed study of cichlids has contributed significantly to the evolution models. To this end, cichlid researchers, in this regard, have adopted a comparative approach that has proven to be an effective tool to elucidate the evolutionary patterns and processes underlying the diversification of this group (Albertson et al., 2005; McMahan et al., 2013; Powder et al., 2015; Woltering et al., 2018). The comparative approach has provided insights into the mechanisms involved in cichlids' evolutionary and adaptative processes (Burress, 2015; Marconi et al., 2023). In particular, the study of cichlids has essential implications for understanding other vertebrates' evolution and informing conservation and management strategies.

The early developmental processes of cichlids, which are renowned for their remarkable diversity and widespread distribution, remain poorly understood. Previous research has predominantly focused on African species (Fishelson, 1995; Fujimura & Okada, 2007; Marconi et al., 2023; Morrison et al., 2001; Powder et al., 2015; Saemi‐Komsari et al., 2018), with scant attention paid to Neotropical species (Beriotto et al., 2023; Kratochwil et al., 2015; Kupren et al., 2014; Meijide & Guerrero, 2000; Molina, 2011; Stephens & Hendrickson, 2001). Further investigations are therefore required to elucidate the complex developmental mechanisms underlying these fascinating group of fishes. Variation in larval morphology and changes in timing, duration, or rate of developmental processes can contribute to detect species‐specific differences, including heterochrony, which can play a crucial role in phenotypic divergence across several groups (Marconi et al., 2023; McKinney & McNamara, 2013). Understanding the ontogenetic development of cichlids is particularly crucial for successfully implementing conservation efforts, because it determines critical stages, elucidates habitat requirements, optimizes reproductive success, recognizes the adaptative strategies, predicts human impacts, and reveals variation in developmental patterns that contribute to genetic diversity (Contreras‐MacBeath et al., 2022; Daly et al., 2021; Karageorgou et al., 2024). Additionally, the socioeconomic impact of cichlid reproduction in fisheries, aquaculture, and the ornamental trade highlights the necessity of comprehending the developmental mechanisms of larvae (Fujimura & Okada, 2007; Giatsis et al., 2015). Moreover, evolutionary studies increasingly incorporate the role of developmental mechanisms, further emphasizing the importance of exploring cichlid ontogeny in greater depth (Marconi et al., 2023; Powder et al., 2015; Woltering et al., 2018). Fish larvae represent the smallest free‐living vertebrates; therefore, they are subject to a range of environmental pressures that limit their capacity to maintain metabolic processes, acquire energy from external sources, and achieve growth (Houde, 2008; Osse & van den Boogaart, 1995). Consequently, the larval stage represents the most vulnerable phase in the life cycle of fishes, as it is characterized by high mortality rates (Gerking, 2014; Sanderson & Kupferberg, 1999). In altricial species, early development is of critical importance as it continues after hatching, and poor nutrition or factors that disrupt development can lead to severe developmental problems, morphological and functional changes, and even mortality (Gerking, 2014; Yúfera et al., 2011; Yúfera & Darias, 2007). The larval stage is defined by two critical phases: initially relying on the yolk sac as an endogenous source of food and later the exogenous feeding while undergoing metamorphosis into the juvenile form (Gerking, 2014; Kendall et al., 1984; Voesenek, 2019). Throughout this stage, fish are typically small and underdeveloped while their sensory, circulatory, muscular, osseous, and digestive systems develop (Kendall et al., 1984). Metamorphosis in fish refers to the transition from the larval stage to the juvenile stage, characterized by the acquisition of phenotypic characteristics typical of an adult (Pinto et al., 2013). It is important to note that marine and freshwater fish species exhibit distinct morphological and functional differences. Among cichlids, those that breed in the substrate (e.g., *Vieja fenestrata*) tend to develop faster than mouth‐brooding species (Gisbert et al., 2014; Lazo et al., 2011).

*V. fenestrata* (Günther, 1860), known as “blackstripe cichlid,” is a cichlid endemic to Mexico that inhabits a variety of aquatic environments in the Atlantic slope from the Papaloapan River basin in the states of Oaxaca and Veracruz in Mexico (Miller et al., 2005). Little is known about the ontogeny of the genus *Vieja* Yépez, 1969, despite their increasing ornamental and aquaculture use (Adamek‐Urbańska et al., 2021; Frías‐Quintana et al., 2021; Sánchez‐Cruz et al., 2024). Therefore, this study aimed to provide a general description of the larval development of *V. fenestrata*. We present an overview of the development from hatching to 15 days post‐hatch. Further, we examine the allometric growth based on eight body proportions. We hypothesized that the ontogenetic development of *V. fenestrata* larvae is characterized by distinct growth patterns, with initial rapid development of sensory and feeding structures followed by subsequent growth shifts to other functional systems. Research on the early ontogeny of fish is of immense significance, as it serves a dual purpose of augmenting the understanding of the developmental features of different species and providing a benchmark for comparison when alterations from typical developmental patterns occur (e.g., chemicals present in the water; Bridi et al., 2017).

## 2 MATERIALS AND METHODS

### 2.1 Breeding of *V. fenestrata*

The *V. fenestrata* broodstock was maintained under laboratory conditions for a minimum of 2 years and spawned regularly at the Laboratory of Rotiferology and Molecular Biology of Plankton at the Universidad Autónoma Metropolitana, Xochimilco (UAM‐X). Adult males and females were subjected to constant conditions (28 ± 1°C, 12:12 light/dark photoperiod cycle) in a 1000‐L tank (200 × 80 × 60 cm). Upon establishing pairs, single pairs were separated into 240‐L tanks (120 × 40 × 50 cm), with only rocks (flagstone) and pots, under the same conditions. The tanks had continuous aeration, mechanical, chemical, and biological filtration, and weekly water changes of 10% of the volume. The fish were fed thrice a day with a commercial omnivorous diet (El Pedregal; 42% protein content). The fish were monitored, and once spawning was completed, the rock carrying the fertilized spawn was transferred to a 40‐L aquarium (50 × 25 × 30 cm) with water from the main aquarium and maintained under the same conditions. The larval development of different clutches was characterized according to the protocol until day 15 post‐hatching. The females of *V. fenestrata* exhibited regular spawning every 16 to 18 days, with a spawning size ranging from 1300 to 1500 eggs, with less than 1% unfertilized eggs. The non‐fertilized eggs and dead embryos were eliminated from the aquariums. Random eggs (*n* = 10) were observed every 3 h under a stereo microscope to follow development until hatching. Embryos’ hatching occurs between 58 and 60 h.

### 2.2 Observation, image acquisition, and measurement

Thirty eggs were collected and measured, and 30 random larvae were similarly collected and observed every 6 h in the first 3 days post‐hatch (dph). Then, observations were made every 12 h after 3 dph. All the observations were conducted on live specimens. Observations described the main morphological and functional features and age recording in hours post‐hatch (hph) and dph, considering hatching as time zero. Image acquisition was performed using a Nikon SMZ1500 stereo microscope equipped with a 5 Mpx resolution camera (Infinity 1, from Lumenera) and illumination provided by a Nikon NI‐30 fiber optic illuminator. The measurements of larvae and eggs were conducted using Image‐Pro Plus v7.1 software (Media Cybernetics, Silver Spring, USA), and images were calibrated using an Olympus 1‐mm calibration ruler. Considering the non‐spherical nature of the eggs, the principal (*Y*) and minor (*X*) axes were measured, and the second minor axes were assumed to be the same length as the first minor axis following the effective diameter equation (*d*_*e*_ = [*Y* + *X* ^2^]^1/3^; Coleman, 1991).

**FIGURE 1:**
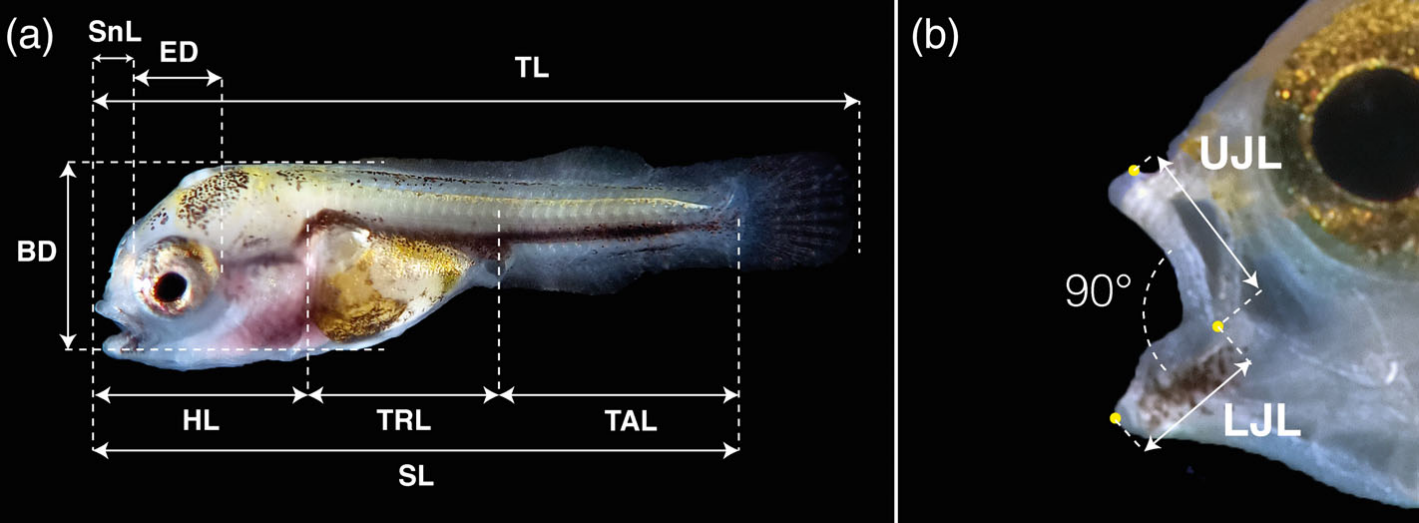
Diagram of morphometric characters measured in *Vieja fenestrata* larvae. (a) Body measurements and (b) mouth measurements. TL, total length; SL, standard length; HL, head length; TRL, trunk length; TAL, tail length; ED, eye diameter; SNL, snout length; BD, body depth; UJL, upper jaw length; LJL, lower jaw length.

Due to the variability in the shape of the yolk sac from day to day, the yolk sac was not measured. The body proportions measured were total length (TL), standard length (SL), head length (HL), trunk length (TRL), tail length (post‐anal; TAL), eye diameter (ED), and body depth (BD; Figure 1a). TL in underdeveloped larvae was defined as the longest linear dimension of the larvae along the anteroposterior axis, and in open‐mouth larvae was defined as the longest linear dimension between the anterior tip of the upper jaw and the posterior end of the caudal fin (Fujimura & Okada, 2007). Furthermore, gape size (GS) was calculated using Shirota's (1970) modified equation proposed by Guma'a (1978): GS = √(UJL^2^ + LJL^2^); by measuring the length from the tip of the upper jaw to the axis of the mouth gape (using upper jaw length [UJL]) and the length from the tip of the dentary to the axis of the mouth gape (lower jaw length [LJL]; Figure 1b). All the measurements (in millimeters) presented in this study are the mean and SD of 15 larvae measured each day (*N* = 255, except for GS—*N* = 150).

### 2.3 Data analysis

The morphometric measurements were analysed in relation to TL using a power function exponent, with the equation *y = ax* ^b^, where *y* is the independent variable (TL), *a* is the intercept, and *b* is the growth coefficient. Isometric growth was indicated by *b* = 1, whereas positive allometric growth occurred when *b*>1, and negative allometric growth occurred when *b* < 1. Student's *t*‐test was utilized to detect differences in isometric *b* values (*b* = 1) at a 95% confidence level. The *t‐*test formula used was *t =* (*b‐b*_*0*_)/S_b_, where *b* is the estimated exponent from the regression, *b*_*0*_ is the hypothesized value, and *S*_*b*_ is the SE of the estimated exponent (Zar, 2010).

The inflection point, where the slope changes, was determined following the methodology outlined by van Snik et al. (1997). Initially, the *x*–*y* dataset was sorted in ascending order based on *x* values (representing TL), and regression analysis was performed based on two distinct segments of the dataset: (1) from the minimum *x* value (*x*_min_) to an intermediate *x* value (*x*_intermediate_) and (2) from the *x*_intermediate_ to the maximum *x* value (*x*_max_). The *x*_intermediate_ value was systematically varied from *x*_min_ + 2 to *x*_max_ − 2 during iterative calculations. *t*‐Tests were performed to assess whether the slopes (*b*) of the regression lines differed significantly between the segments (*x*_min_ to *x*_intermediate_ and *x*_intermediate_ to *x*_max_). The *x*_intermediate_ value corresponding to the largest *t*‐value, indicating the most significant difference in slopes, was defined as the inflection point. It is important to note that *t*‐tests were solely used for identifying the inflection point and were not reported in the results.

### 2.4 Ethics statement

All procedures were performed adhering to strict standards for animal care and research set by the Mexican government (NOM‐062‐ZOO‐1999) and “The International Council for Laboratory Animal Science (ICLAS).”

## 3 RESULTS

**FIGURE 2:**
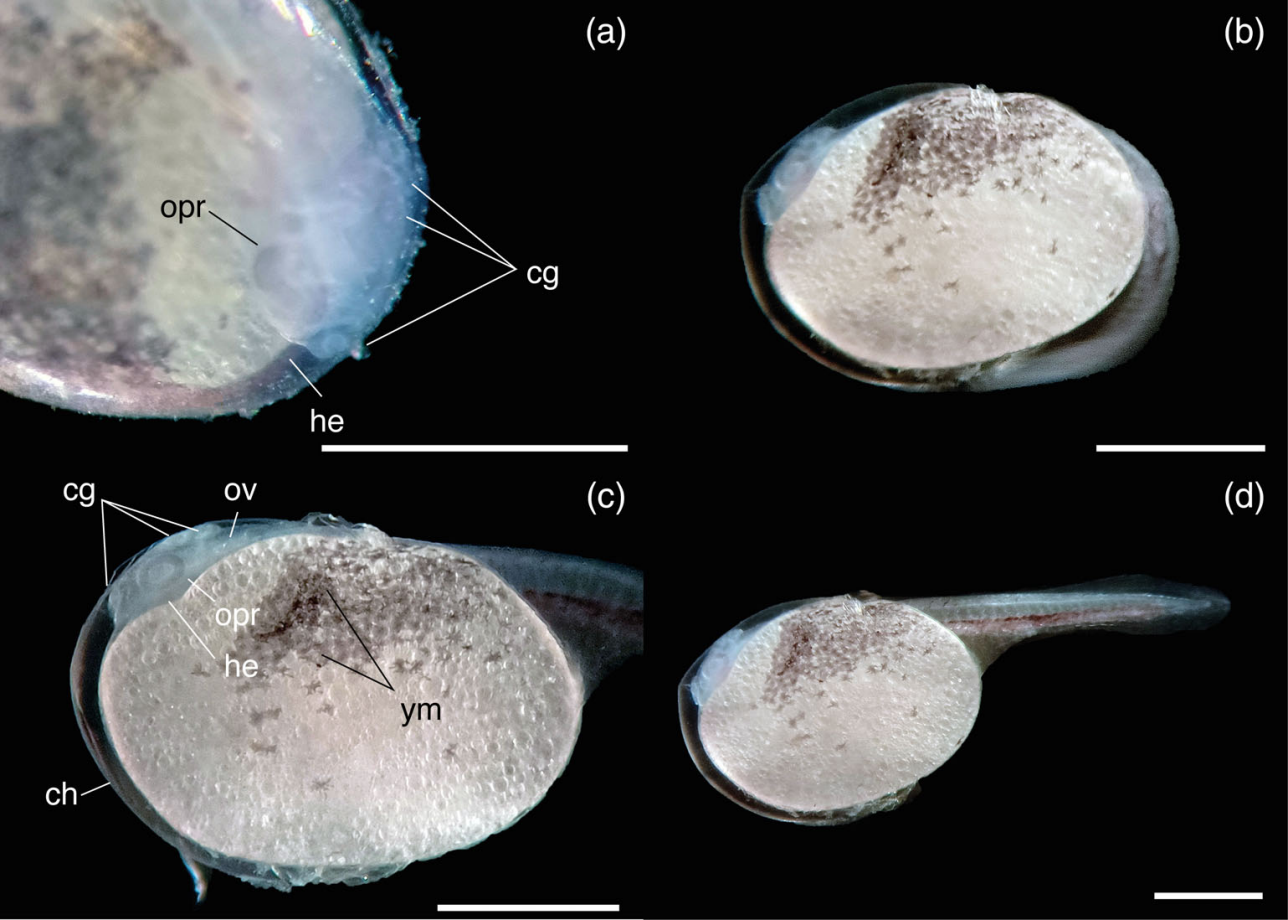
Hatching of *Vieja fenestrata* larvae. cg, cement glands; ch, chorion; he, heart; opr, optic primordium; ov, otic vesicle; ym, yolk melanophores. Scale bar = 1 mm. (a) Larvae before hatching; (b) larvae hatching; (c) larvae with anterior part inside the egg; (d) larvae partially emerging from the chorion.

**FIGURE 3:**
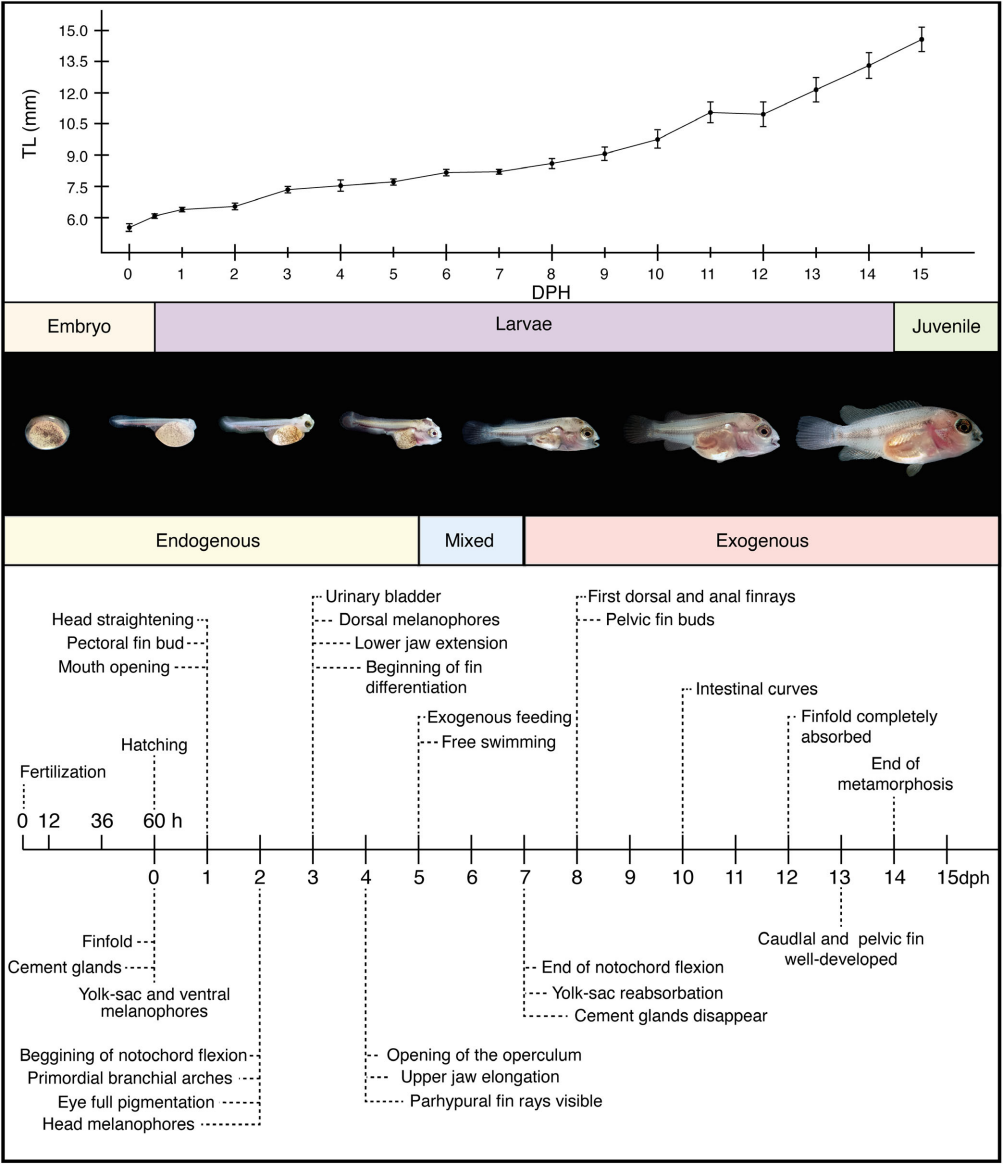
Main events during larval development and growth of *Vieja fenestrata*.

*V. fenestrata* exhibits substrate‐spawning behavior characterized by the attachment of its elliptical telolecithal eggs on a substrate via an adhesive mucous layer (*d*_*e*_ = 1.94 ± 0.06 mm; see Figure 2a). The eggs are meroblastic and display asynchronous hatching between 58 and 60 h postfertilization, with larvae emerging from the egg a few minutes apart. Notably, the posterior part of the body is the first to hatch (Figure 2b,c). In contrast, the anterior part remains inside the egg (Figure 2c,d). After a couple of minutes, the larvae emerge entirely from the chorion, continuing to move actively. The changes in the TL and significant developmental events of *V. fenestrata* are illustrated in Figure 3.

### 3.1 Morphological development

The following sections delineate the principal events for the different developmental stages:

#### 3.1.1 0HPH

**FIGURE 4:**
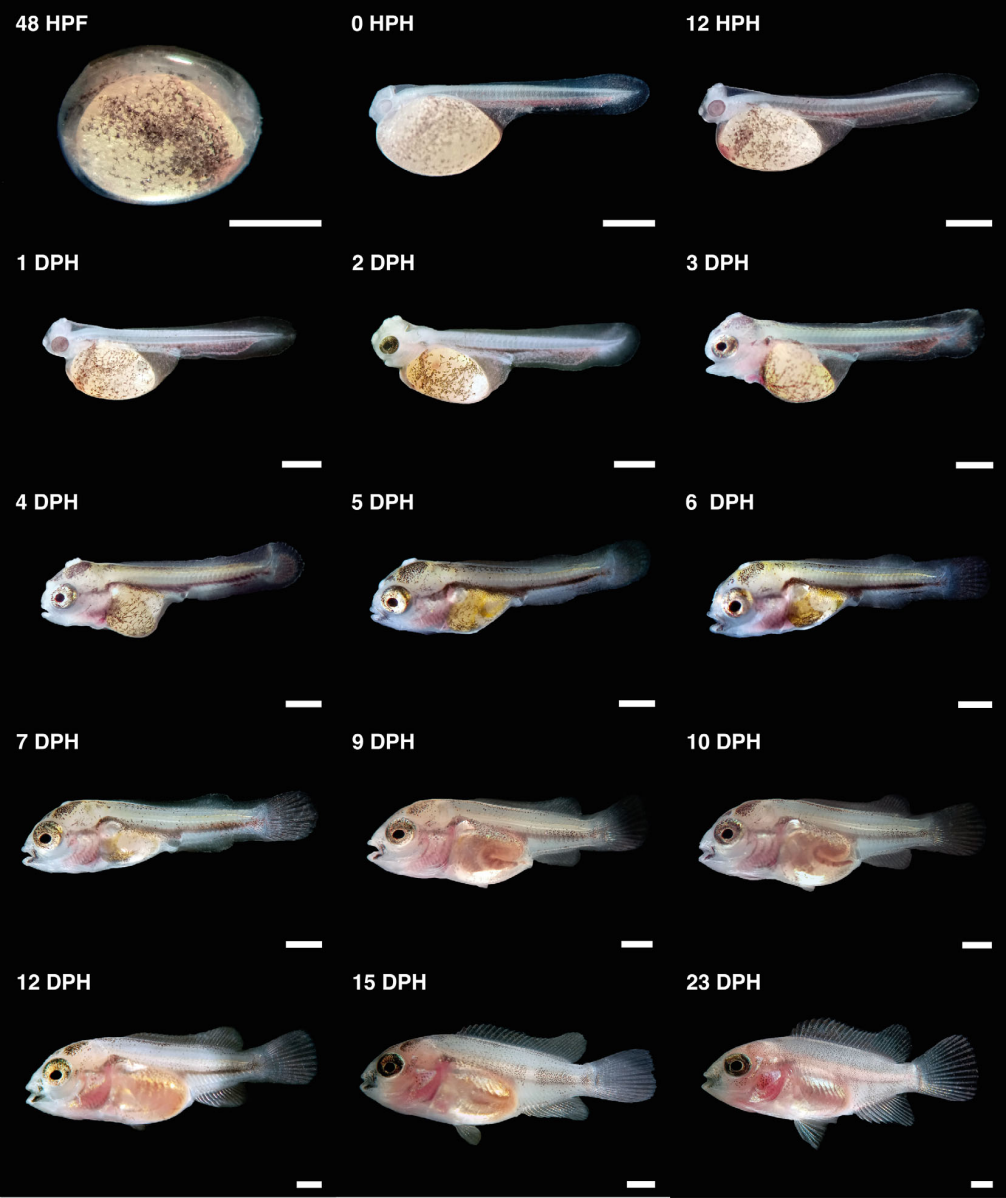
Overview of the larval development of *Vieja fenestrata*. Scale bars = 1 mm.

**FIGURE 5:**
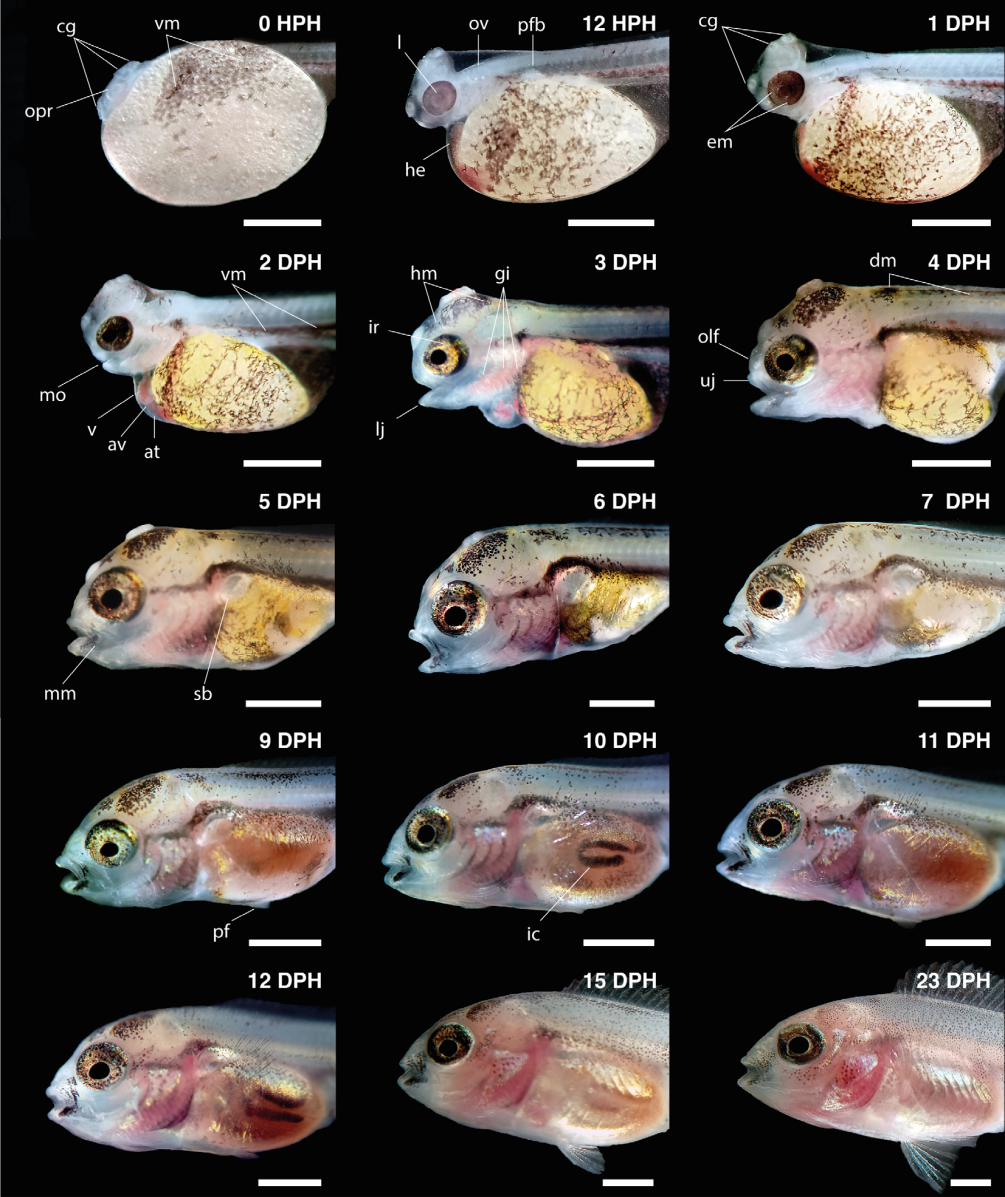
Head development in larval development of *Vieja fenestrata*. Lateral views of the living larvae. av, atrioventricular valve; at, atrium; cg, cement glands; dm, dorsal melanophores; em, eye melanophores; g, gills; he, heart; hm, head melanophores; ic, intestinal curves; ir, iridophores; l, lens; lj, lower jaw; mm, mouth melanophores; olf, olfactory organ; opr, optic primordium; ov, otic vesicle; pf, pectoral fin; pfb, pectoral‐fin bud; sb, swim bladder; uj, upper jaw; v, ventricle; vm, ventral melanophores; ym, yolk melanophores. Scale bar = 1 mm.

The newly hatched larvae measured 5.52 ± 0.19 mm TL (Figure 4), with a large yolk sac (>60% of the body). Larvae have a single finfold covering the body from the dorsal area of the trunk to the ventral area of the yolk sac. The notochord extends from the hindbrain line to the tail bud straight at the end. The head is slightly differentiated. Optic primordium and lens were visible at 0 hph. The colorless eye increased pigmentation after 6 hph. An otic vesicle with two otoliths is visible in the posterior head region. Cement glands were in pairs: two dorsal glands (parietal area) and one ventral gland (frontal region). Larvae formed groups of 8 to 12 individuals attached to form a crown‐like aggregation at the bottom of the tanks. Particulate organic matter, fibers, and remnants of chorion were also found stuck to the glands. The heart is visible in a tube‐shaped form. The heart blood vessels, the yolk‐sac capillaries, and the plexuses in the ventral finfold are red colored (Figure 4). Ventral melanophores are present, forming a stripe throughout the body and scattered over the yolk sac. The digestive tract and anus are visible. After 12 hph, the head was straightening (TL = 6.08 ± 0.11 mm). The yolk sac at hatching is elliptical, but at this time, it becomes wider in the ventral part of the trunk and narrower with a cone‐like tip at the posterior end (Figure 5).

#### 3.1.2 1DPH

(TL = 6.39 ± 0.10 mm). At 1 dph, the pectoral‐fin bud is visible, and the notochord is still straight. The head continued to straighten and lift off the yolk sac. The eyes have pigmentation and appear gold due to iridophores. Cement glands have become prominent. The ventral finfold vascularization and peripheral circulation have increased, along with larvae moving vigorously. The heart is visible in the anterior of the yolk sac, and the ventricular and atrial chambers are distinguishable. The mouth has not formed yet, but it appears to be open.

#### 3.1.3 2DPH

**FIGURE 6:**
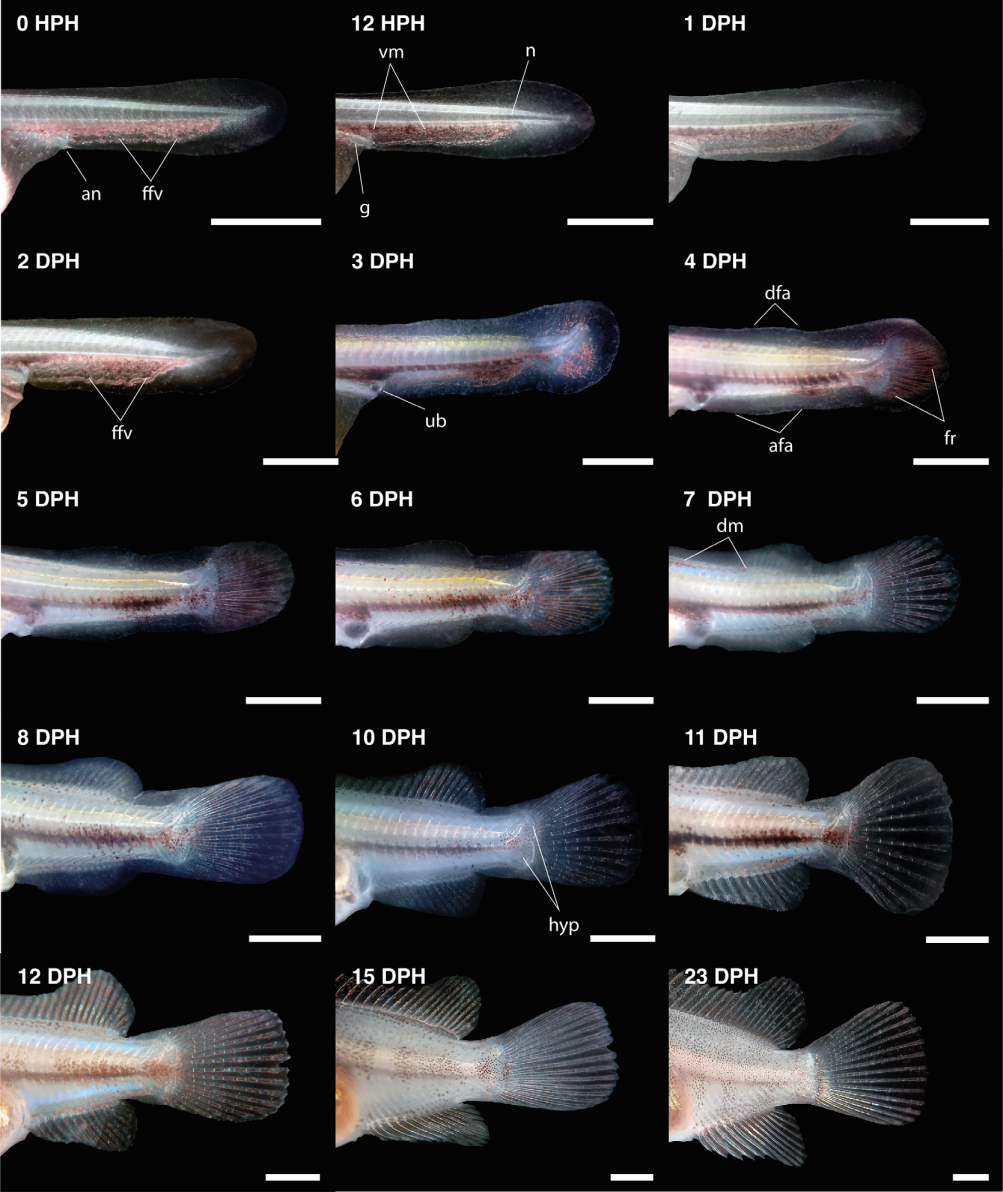
Lateral views of the tail region of *Vieja fenestrata* larvae during larval development. afa, anal‐fin anlage; an, anus; dfa, dorsal‐fin anlage; dm, dorsal melanophores; ffv, finfold veins; fr, fin rays; g, gut; hyp, hypural; n: notochord; ub, urinary bladder; vm, ventral melanophores. Scale bar = 1 mm.

(TL = 6.53 ± 0.16 mm). The larvae have developed a straight head. The cement glands have grown in size and shifted slightly. The eyes have a golden ring around the lens. The digestive tract continues to develop and shows peristaltic movements. The notochord began to flex. The caudal tip has rays primordia with increased vascularization (Figure 6). The pericardial cavity has increased in size, and the heart is developing. Branchial arches primordia are visible.

#### 3.1.4 3DPH

(TL = 7.34 ± 0.16 mm). During this day, the caudal fin is transformed from a flat plane into a bud‐like structure with a rounded shape, and the notochord continues to flex. The branchial arches become more differentiated, and gill filaments begin to form. The dorsal and anal fins start to differentiate, and rays primordia appear in the pectoral fin. Ventral finfold vascularization decreases and becomes less visible. The lower jaw extends anteriorly, and new melanophores appear in the dorsal part of the body. Additionally, the urinary bladder can be easily distinguished near the digestive tract.

#### 3.1.5 4DPH

(TL = 7.54 ± 0.27 mm). During this day, the larvae attempted swimming. However, they could only make short‐term movements due to their inability to float completely. This limitation may be due to the size of the yolk sac. The movements of the gills and mouth are much stronger, along with the first eye movements, and the operculum opens. The upper jaw also elongated during this period. The caudal fin rays became more developed and were arranged in two segments, with the parhypural fin rays visible.

#### 3.1.6 5DPH

(TL = 7.54 ± 0.27 mm). The larvae started to swim freely and feed exogenously without exhausting the yolk sac (mixed feeding). Jaw extension was observed, and the cement glands became smaller and diffused. The inflation of the swim bladder began and could be distinguished (Figure 5).

#### 3.1.7 6–14 DPH

At 6 dph (TL = 8.16 ± 0.15 mm), the swim bladder began to enlarge while iridophores appeared laterally to the gut and on the operculum surface. By 7 dph (TL = 8.22 ± 0.10 mm), the cement glands were regressing and eventually disappeared. At this point, the larvae had exhausted their yolk sacs entirely and finished notochord flexion. Dorsal‐fin rays started to emerge and develop while pelvic‐fin buds were also visible. The first dorsal‐ and anal‐fin rays became distinguishable by 8 dph (TL = 8.60 ± 0.25 mm; see Figure 6). Intestinal curves could be seen by 10 dph (Figure 5). By day 12, the anal and dorsal fins had reached the end of the caudal peduncle, and the finfold was absorbed entirely, forming the caudal peduncle. At 14 dph, the larvae took on a juvenile form similar to adults, marking the end of the metamorphosis. Pigmentation continued in juveniles, with melanophores increasing and being arranged along the trunk, tail, and fins to form dark blotches (see Figure 5).

### 3.2 Larval growth

**FIGURE 7:**
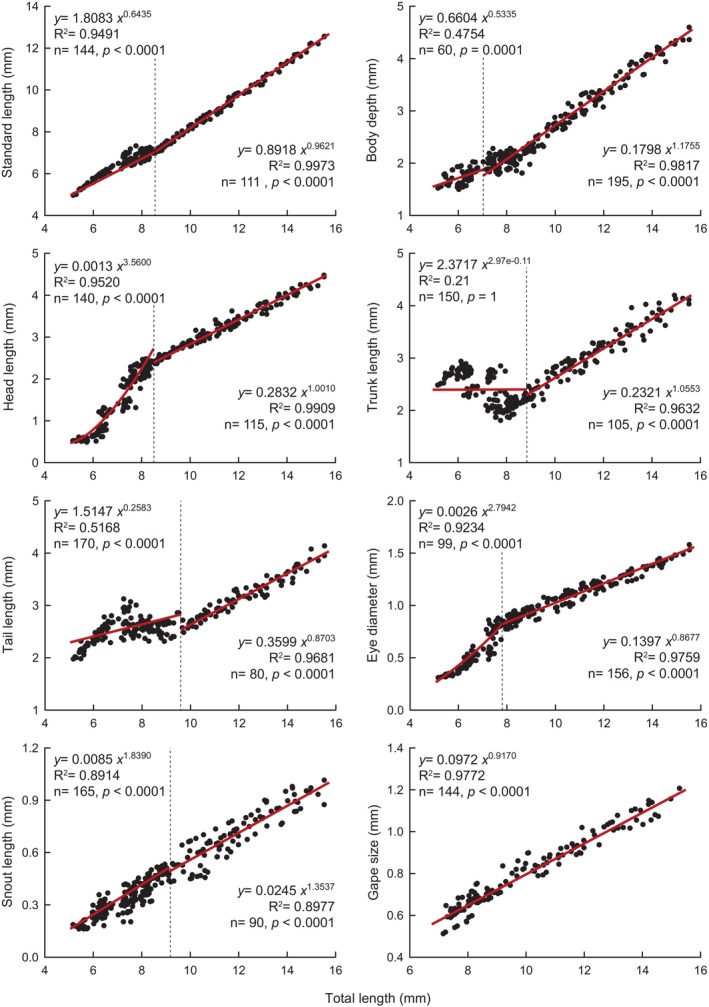
Relation between measured morphological proportions and total length during the larval stage of *Vieja fenestrata*. The dashed line represents the growth inflection point.

The morphological proportions of *V. fenestrata* underwent substantial changes during the larval period (see Figure 7). The growth of GS demonstrates a near isometric pattern (*b* = 0.92; *p =* 0.02) without displaying an inflection point, whereas other proportions exhibited inflection points. Before the inflection point, negative allometric growth was observed for TAL, TRL, and BD (*b* < 0.55; *p <* 0.0001), whereas positive allometric growth was observed for head morphological proportions: HL, ED, and SNL (*b =* 3.56, 2.79, and 1.84, respectively; *p <* 0.0001). After the inflection point, positive allometric growth was observed for BD and SNL (*b* = 1.18 and 1.35, respectively; *p* < 0.001); HL (*b* = 1.00; *p =* 0.938) and TRL (*b =* 1.06; *p* < 0.062) exhibited isometric growth. Conversely, TAL and ED displayed negative allometric growth (*b* = 0.87 and 0.86, respectively; *p* < 0.001). Notably, HL, ED, and SNL witnessed a reduction in growth coefficients after the inflection point (*b* = 3.56 vs. 1.00; 2.79 vs. 0.87; 1.84 vs. 1.35, respectively).

## 4 DISCUSSION

This study describes the larval development and growth patterns of *V. fenestrata*. In some fishes, organogenesis occurs during the embryonic stage before hatching (precocial), whereas others undergo organ differentiation after hatching during the metamorphosis (altricial; Balon, 1981). *V. fenestrata* are born altricial with poorly developed bodies. During early ontogeny, *V. fenestrata* larvae undergo several morphological, physiological, and behavioral changes that include the development of respiratory, feeding, and locomotory mechanisms (Figure 3) that are like those found in larvae of other Neotropical substrate spawners cichlid species (Beriotto et al., 2023; Kratochwil et al., 2015; Meijide & Guerrero, 2000; Molina, 2011). The first 5 dph are characterized by significant changes related to first exogenous feedings, such as eye pigmentation, mouth opening, jaws and pectoral‐fin development, and swim bladder inflation (Figure 5). Previous research has demonstrated a close relationship between the timing of mouth opening and yolk‐sac absorption (Lasker et al., 1970). The start of exogenous feeding is a crucial stage in the development of larvae, as it marks the transition from endogenous nutrient sources to exogenous sources. This phase plays a pivotal role in the growth and survival of larvae (Yúfera & Darias, 2007). *V. fenestrata* exhibits a mixed feeding period from 5 to 7 dph, gradually shifting from yolk‐sac to exclusively exogenous feeding. Notably, during this stage, vitellus can still serve as a backup nutrient source while the larvae learn to feed exogenously or in case of food scarcity (Balon, 1986; Jaroszewska & Dabrowski, 2011). The increase in behavioral abilities (encounter, attack, capture, ingestion, rejection, and escapes), swim capacity, and performance of the larvae is crucial for maximizing food acquisition (Rønnestad et al., 2013).

During the first 2 dph, the ventral finfold of larvae demonstrated robust vascularization, resulting in increased peripheral circulation coupled with vigorous larval movements (Figure 6). It has been established that oxygen uptake predominantly occurs through cutaneous routes in larvae (Fu et al., 2010; Rombough, 2002). Consequently, the primordial finfold is believed to enhance respiration and locomotion by expanding the surface area, whereas the pectoral fins promote water flow across the skin in larvae (Osse & Van den Boogaart, 1995; Zimmer et al., 2020). However, the vascularization of the ventral finfold decreases and becomes less visible after 3 dph, presumably due to the shift from cutaneous respiration to gill respiration (Figure 5). The sequence of fin formation in *V. fenestrata* is consistent with that of other Neotropical species, comprising the caudal, dorsal, anal, pelvic, and pectoral fins (Beriotto et al., 2023; Kratochwil et al., 2015; Meijide & Guerrero, 2000; Molina, 2011). By 12 dph, the anal and dorsal fins had reached the end of the caudal peduncle, and the finfold was absorbed entirely, forming the caudal peduncle. The development of the caudal fin and its supporting elements facilitates the larvae's initial swimming movements and more active displacement in the water column, which is critical for exogenous feeding.

*V. fenestrata* possesses an adhesive mechanism during its larval stage with six cement glands. These glands comprise two dorsal glands located in the parietal area and one ventral gland in the frontal region. However, the cement glands undergo regression, eventually disappearing by the seventh dph (Figure 5). In aquatic vertebrate larvae, the presence of an adhesive organ that secretes sticky mucus is a common phenomenon (Picard, 1976). Teleosts, including several cichlid species, possess specialized papillae for larval attachment (Groppelli et al., 2003; Kratochwil et al., 2015). These papillae are composed of mucous‐secreting collocytes that mediate the attachment process. However, these structures begin to disappear once the larvae can swim or their yolk sac is depleted (Groppelli et al., 2003; Nelson et al., 2019; Pottin et al., 2010). *V. fenestrata* larvae tend to form groups of 8 to 12 individuals, which attach to form a crown‐like aggregation at the bottom of the tanks. A similar behavior has been reported for *Pelmatolapia mariae* (Boulenger, 1899) (Pottin et al., 2010). The adhesive mechanism and larval aggregation behavior observed in *V. fenestrata* and other cichlid species could be related to avoiding predation and withstanding currents in lotic environments.

The most pronounced morphological and morphometric changes (inflection points) for *V. fenestrata* occurred between 3 and 8 dph. The larvae's growth coefficient indicated that body parts' growth differed at each developmental stage, thereby supporting the hypothesis that ontogenetic priorities change during development to enhance survival probability (Osse et al., 1997; Osse & van den Boogaart, 1995). The transition from larval to juvenile form is marked by important changes in the head, trunk, and tail segments exhibiting different allometric growth (Khemis et al., 2013; Osse et al., 1997). These changes comprise several crucial anatomical and physiological transformations focusing on sensory organs, the skeleton, the mouth, muscle mass, and the digestive system. During larval development, *V. fenestrata* showed a positive allometric growth in HL from hatching to the inflection point, followed by isometric growth; TRL changed from a negative allometric growth before the inflection point to an isometric growth; and TAL displayed a negative allometric growth throughout development. As shown in Figure 4, the tail segment underwent several changes during fin development associated with locomotion function and exhibited less growth compared to the other segments. The negative allometric growth in TRL before the inflection point might be related to the yolk sac, followed by isometric growth associated with the development of organs and tissues related to locomotion and metabolism (Saemi‐Komsari et al., 2018). Head development in *V. fenestrata* showed the highest positive allometric patterns from hatching to the inflection point. The development of the head reflects the priority maturation of organs and systems associated with crucial functions, including sensory, nervous, and feeding mechanisms in the head region, all of which are critical for successful exogenous feeding (Fuiman, 1983; Osse & van den Boogaart, 1995). This development enables the larvae to respond to light stimuli, locate and capture prey, detect predators, and transition from relying on endogenous to exogenous feeding, thus increasing growth rates and improving survival probabilities during early ontogenetic stages (Gisbert et al., 2002; Peña & Dumas, 2009). Another important feature related to the transition from endogenous to exogenous feeding is the GS, which is crucial for food selection, capture, handling, and consumption (Goatley & Bellwood, 2009; Makrakis et al., 2008; Yúfera & Darias, 2007). Although the GS presented a negative allometry, it increased in size during the larval development of *V. fenestrata*. As fish grow, they tend to consume a wider range of food sizes, with the average prey size increasing (Arim et al., 2010; Yúfera et al., 2011). This increase in prey size is attributed to both morphological changes, such as ontogenetic increases in GS, and physiological improvements, such as enhanced vision and swimming abilities (Wootton, 1998).

Many morphological characters display relatively fast growth in early development followed by a slowdown of growth and tend to be isometric (Oikawa & Itazawa, 1985). In older larvae and juveniles, all coefficients approach 1, indicating a stabilization of relative growth priorities, signifying the fulfillment of primary functions during the early developmental stages and the attainment of relative growth stability (Fuiman, 1983; Osse & van den Boogaart, 1995; Osse & van den Boogart, 2004; Pinder & Gozlan, 2004; van Snik et al., 1997). This slowdown pattern was observed after the inflection point in SNL (remain positive allometric growth), HL (isometric), and the most noticeable in ED (negative allometric). Furthermore, the pattern of positive allometry followed by negative allometry in ED is consistent with findings in other species (Kupren et al., 2014; Xu et al., 2023). This is evidenced by the accelerated development of the eye during the first days until reaching a certain maturity (see Figure 5) followed by a stabilization in growth. The positive allometry for BD in *V. fenestrata* may be associated with the transition from larval to juvenile form and the development of internal organs (e.g., digestive tract and swim bladder).

Despite the ecological importance of cichlid species, more attention needs to be given to their fundamental biological aspects, particularly the development of their larvae. A new approach that focuses on studying variation in early cichlid ontogeny has been proposed (Kratochwil et al., 2015; Marconi et al., 2023; Parsons et al., 2014; Powder et al., 2015; Prazdnikov & Shkil, 2019). As a significant proportion of morphogenetic processes occur during embryonic and larval development, studying variation in early ontogeny is crucial for understanding when and how divergent phenotypes form (Marconi et al., 2023). One of the most common differences between species and within species is the variability in the timing, rate, and duration of specific developmental events. These differences are believed to arise from variations in embryonic and larval morphology and changes in the timing, duration, or rate of developmental processes, such as heterochrony. Heterochrony is known to cause or contribute to phenotypic divergence (Bird & Webb, 2014; Dobreva et al., 2022; Holtmeier, 2001; Marconi et al., 2023). Therefore, it is essential to understand the variation in early ontogeny of cichlids to elucidate the mechanisms underlying phenotypic variation and divergence. Further studies might focus on particular characters during the early ontogeny of cichlids, such as skeletal ontogeny (Beriotto et al., 2023; Marconi et al., 2023); digestive feeding apparatus and digestive enzyme activities (Adamek‐Urbańska et al., 2021; Albertson & Kocher, 2006; Cuenca‐Soria et al., 2013; López‐Ramírez et al., 2010), and colouration patterns (Hilsdorf et al., 2002; Marconi et al., 2023; Prazdnikov & Shkil, 2023).

Middle‐American cichlids, particularly those in the Herichthyini clade, hold significant ecological, evolutionary, and biogeographic importance. Comprising approximately 50 species, predominantly distributed in the northern parts of Middle America (Alda et al., 2021; McMahan et al., 2015; Rico et al., 2022), for these fishes species make noteworthy contributions to the area's biodiversity. They have been extensively studied to unravel the evolutionary patterns and biogeographic trends shaped by Middle America's geological history, physiography, and topography (Alda et al., 2021; Matamoros et al., 2012; Matamoros et al., 2014; McMahan et al., 2015; Mejía et al., 2022; Říčan et al., 2013). Despite the smaller size of Middle America compared to tropical South America, it boasts a remarkably diverse cichlid fauna, with almost all species belonging to the Heroini lineage (Kullander, 2003; Říčan et al., 2016). These cichlids exhibit morphological and ecological diversities, as a result of different factors, including feeding habits and habitat preferences. Furthermore, cichlids have significant cultural, economic, and nutritional value in native Mexican communities, where they, along with other freshwater fish species, serve as vital protein sources through both artisanal and commercial food fisheries (Contreras‐Balderas et al., 2008; Lyons et al., 2020). However, overharvesting and introducing nonnative species pose substantial threats to natural populations. Sustainable management practices are critical to safeguard native fish populations, as overfishing has been identified as one of the primary factors impacting species numbers (Lyons et al., 2020). Information about the basic biology, such as the species' larval development and nutritional aspects, is necessary to bring native species to production in captivity (Dávila‐Camacho et al., 2019). Within the herichthyinis, the genus *Vieja* has aroused considerable interest for its production in captivity in aquaculture (Adamek‐Urbańska et al., 2021; Frías‐Quintana et al., 2021; Sánchez‐Cruz et al., 2024). Therefore, further research is required to assess the potential for sustainable fish farming practices that can support the conservation of these species while also providing economic benefits to local communities.

These findings can help us better understand and emphasize the adaptative strategies of the species. The larvae of *V. fenestrata* hatch with underdeveloped bodies but undergo rapid morphological, physiological, and behavioral changes. Different body parts grow at varying rates during different stages, indicating shifting developmental priorities that contribute to the survival. For example, there is a rapid development of the head to improve sensory perception and feeding efficiency followed by changes in growth patterns to meet evolving functional needs. As the larvae transition from relying on the yolk sac to exogenous feeding, there is a brief mixed period during which the yolk sac serves as a backup nutrient source. Early development of eyes, mouth, jaws, and pectoral fins is crucial for initiating exogenous feeding, and as the larvae develop, they transition from cutaneous respiration facilitated by a well‐vascularized ventral finfold to gill respiration. The observed behavioral adaptations include larval aggregation, an adhesive mechanism, and increased swimming capacity, which collectively reduce the risk of predation, help the larvae withstand currents, improve food acquisition, and help to avoid predators.

## 5 CONCLUSIONS

The study of developmental ontogeny in *V. fenestrata* larvae provides valuable insights into its life history. The species displays substrate‐spawning behavior, whereby elliptical telolecithal eggs are attached through an adhesive mucous layer. Asynchronous hatching occurs between 58 and 60 h, with the posterior body emerging first. Early developmental stages are characterized by significant morphological, physiological, and behavioral changes over 14 days. The larvae exhibit distinctive features such as a large yolk sac, single finfold, and visible optic and otic structures upon hatching. Notable changes in morphology and behavior mark progression in larval development. The larvae show increased vascularization, fin differentiation, and heightened activity within the first day. Subsequent days witness further morphological refinements, including flexion of the notochord, differentiation of fins, and emergence of swim bladder functionality. This species undergoes a mixed feeding period from 5 to 7 dph, gradually shifting from yolk‐sac to exclusively exogenous feeding. Over the following days, absorption of the yolk sac, regression of cement glands, and development of fin rays signify advanced maturation. Metamorphosis is completed within 14 dph, marking the transition from larvae to juvenile stage. Throughout development, pigmentation patterns evolve, and structures necessary for survival and locomotion develop gradually. These findings shed light on the intricate developmental process of *V. fenestrata*, underscoring the interplay of morphological changes and behavioral adaptations that are crucial for survival and the eventual transition to the juvenile stage. Further studies may explore the ecological implications of these developmental dynamics to elucidate evolutionary implications.

## AUTHOR CONTRIBUTIONS

Rubén Alonso Contreras‐Tapia: Conceptualization, formal analysis, investigation, methodology, writing—original draft, writing—review and editing, and visualization. Marcela Ivonne Benítez‐Díaz Mirón: Formal analysis, data curation, writing—review and editing, visualization. Gabriela Garza Mouriño: Conceptualization, methodology, writing—review and editing. María Elena Castellanos‐Páez: Conceptualization, methodology, writing—review and editing. All authors read and approved the final manuscript.

## FUNDING INFORMATION

This research received no specific grant from any funding agency in the public, commercial, or not‐for‐profit sectors.

## CONFLICT OF INTEREST STATEMENT

The authors declare no conflict of interest.

## Data Availability

The data that support the findings of this study are available from the authors upon reasonable request.
